# The second international conference on reducing burden of traffic accidents, Shiraz, Iran

**DOI:** 10.5249/jivr.v5i1.322

**Published:** 2013-01

**Authors:** Kamran B.Lankarani, Mojtaba Mahmoodi, Sayed Taghi Heydari, Hassan Joulaei, Fariborz Ghaffarpasand, Najmeh Maharlouei, Mohammad Reza Aghabeigi, Ghasem Moafian, Navid Yamini, Arman Najafi

**Affiliations:** ^*a,*^Health Policy Research Centre, Shiraz University of Medical Sciences, Shiraz, Iran.

Road crashes kill nearly 1.3 million people every year, and leave millions moreinjured or permanently disabled. Impaired driving, unsafe roads and other dangersshatter lives in a matter of seconds.

The Decade can help thwart this needless loss of life.

“I call on Member States, international agencies, civil society organizations, businesses and community leaders and people everywhere to ensure that the decade leadsto real improvements.” Mr. Ban Ki-moon, United Nations (UN) Secretary-General.^1^

According to the World Health Organization (WHO) report, mortality from traffic accidents have increased from almost 999,000 in 1990 to more than one million in 2002 and is projected to approach 2 million death annually by 2020.^2^ While only 40% of the world’s motor vehicles are owned by inhabitants of developing countries, 85% of total mortality of traffic accidents occur in these countries.^3,4^ This decade from 2011 to 2020 has been called as the “Decade of Action” for road safety by World Health Organization (WHO).^5-7^ This is an opportunity to have more intersectoral collaboration for this important goal both within and between countries. In concordance with the UN announcement of “Decade of Action for Road Safety 2011-2020”, and for promoting a collaborative action for reducing traffic accidents’ burden, Health Policy Research Centre, organized this international conference for second time.^8^

**Authorities and Participants**

The second international conference on reducing burden of traffic accidents; challenges and strategies was held by Health Policy Research Centre affiliated with Shiraz University of Medical Sciences, Shiraz, Iran in collaboration with several other organizations and agencies including Iranian Police, Iranian emergency medical services, Iranian Forensic Medicine Organization and Oklahoma State University, (Oklahoma, USAon 2012 March 10th and 11th at Velayat Conventional Centre, Shiraz, Iran. The main themes of the conference were evidence based review of:

• Interventions and projects to reduce traffic accidents 

• Environmental determinants of traffic accidents 

• Enhancing safety of pedestrians and road users

• Comprehensive traffic accident registration 

• Socio-cultural factors affecting traffic accidents 

**Sessions and Speeches**

Overall 350 abstracts in different subjects of traffic accidents were sent to the seminar secretariat out of which 123 were selected by scientific board of the conference after peer review for presentation (26 oral and 97 poster presentations). 

During the congress representative form WHO regional office at the Eastern Mediterranean presented the WHO strategy for the policy formulation at national level as well as how road traffic accidents become a global health concern during recent years. She also stated that road safety is a shared responsibility between government and legislative bodies, media, professionals, NGOs(and special interest groups), police, industry, health, users and citizens. The head commander of the traffic sector of the police headquarter stated that the number of cars in Islamic Republic of Iran will reach 15 million at the end of 2011. This number was 4 million and 8 million by the years of 2001 and 2005 respectively and it will exceed 30 million by end of this decade. The importance of utilizing structured guidelines in the management of victims of traffic accidents was emphasized in this conference.

The role of infrastructures in developing countries in increased accidents and their burden in developing countries was highlighted in several presentations. The psychology of response to accidents and the way they could be promoted also was discussed in this meeting. 

Challenges of trauma care centers and emergency medical services in handling traffic accidents was the theme of a panel of physicians, surgeons and EMS technicians in collaboration with representatives from Fars governor. It was stressed thatcoordinated teamwork is the most needy action to increase the survival of the multiple trauma patients. Policies and barriers of trauma research were also addressed. One of the important aspects of this conference was on line launching of the unified, comprehensive traffic accident registry in Shiraz metropolitan. This project was started two years before by the Health Policy Research Center, Shiraz University of Medical Sciences, Shiraz, Iran. 

**Take-home Message**

Traffic injuries are among emerginghealth issues. Coordinated regional, national and international dynamic plan is needed to meet therequirements of developing effective and timely policies in pre-injury, injury and post-injury phases. These policies should be based on best available evidences. But all policies should be practical. This practicality is related to consideration of available resources, cultures, intersectoral collaboration and more involvement of community.

**Acknowledgments**

We thank all the staff members of Health Policy Research Centre, Shiraz, Iran for their kind cooperation and collaboration for holding the conference.
